# Predicting severe radiation-induced oral mucositis in head and neck cancer patients using integrated baseline CT radiomic, dosimetry, and clinical features: A machine learning approach

**DOI:** 10.1016/j.heliyon.2024.e24866

**Published:** 2024-01-24

**Authors:** Razieh Agheli, Zahra Siavashpour, Reza Reiazi, Samira Azghandi, Susan Cheraghi, Reza Paydar

**Affiliations:** a Radiation Sciences Department, Faculty of Allied Medicine, Iran University of Medical Sciences, Tehran, Iran; b Department of Radiation Oncology, Shohada-e Tajrish Educational Hospital, Shahid Beheshti University of Medical Sciences, Tehran, Iran; c Department of Radiation Physics, Division of Radiation Oncology, University of Texas MD Anderson Cancer Center, Houston, TX, 77030, USA; d Radiation Biology Research Center, Iran University of Medical Sciences, Tehran, Iran

**Keywords:** Radiomics, Machine learning, Toxicity prediction, Oral mucositis, Head and neck cancer

## Abstract

**Purpose:**

To establish the early prediction models of radiation-induced oral mucositis (RIOM) based on baseline CT-based radiomic features (RFs), dosimetric data, and clinical features by machine learning models for head and neck cancer (HNC) patients.

**Methods:**

In this single-center prospective study, 49 HNCs treated with curative intensity modulated radiotherapy (IMRT) were enrolled. Baseline CT images (*i.e.,* CT simulation), dosimetric, and clinical features were collected. RIOM was assessed using CTCAE v.5.0. RFs were extracted from manually-contoured oral mucosa structures. Minimum-redundancy-maximum-relevance (mRMR) method was applied to select the most informative radiomics, dosimetric, and clinical features. Then, binary prediction models were constructed for predicting acute RIOM based on the top mRMR-ranked radiomics, dosimetric, and clinical features alone or in combination, using random forest classifier algorithm. The predictive performance of models was assessed using the area under the receiver operating curve (AUC), accuracy, weighted-average based sensitivity, precision, and F1-measure.

**Results:**

Among extracted features, the top 10 RFs, the top 5 dose-volume features, and the top 5 clinical features were selected using mRMR method. The model exploiting the integrated features (10-radiomics + 5-dosimetric + 5-clinical) achieved the best prediction with AUC, accuracy, sensitivity, precision, and F1-measure values of 91.7 %, 90.0 %, 83.0 % 100.0 %, and 91.0 %, respectively. The model developed using baseline CT RFs alone provided the best performance compared to dose-volume features or clinical features alone, with an AUC of 87.0 %.

**Conclusion:**

Our results suggest that the integration of baseline CT radiomic features with dosimetric and clinical features showed promising potential to improve the performance of machine learning models in early prediction of RIOM. The ultimate goal is to personalize radiotherapy for HNC patients.

## 1 Introduction

Radiotherapy (RT) is an integral component of the multidisciplinary approach in the treatment of head and neck cancer (HNC) patients [[1], [2], [3]]. Although RT is an effective primary treatment modality for HNC, it often induces numerous life-altering morbidities which influence the quality of life (QOL) of the patients [4], of which oral mucositis (OM) is one of the most prevalent acute complications [[5], [6], [7]], accounting for more than 90 % of HNC patients treated with standard regimens [6]. Radiation-induced oral mucositis (RIOM), an inflammatory or ulcerative lesion, is initiated by incidental irradiation to basal epithelial cells [7,8]. RIOM can result in severe pain in the oropharynx, dysphagia, language disorder, and weight loss; and therefore, decreased QOL [7]. RIOM can also lead to the interruption of RT fractions and is a major dose-limiting factor in accelerated, dose-escalated RT regimens designed to improve the likelihood of tumor control [9,10].

Modern RT techniques, *e.g.,* intensity-modulated radiotherapy (IMRT), have outperformed the conventional RT techniques, in attempting to alleviate radiation-induced toxicities [11]. Nonetheless, it has been reported that IMRT does not decrease the incidence of RIOM; especially in patients with nasopharyngeal cancer (NPC) [6]. Accurate prediction of RIOM can assist clinicians for early intervention. There are some guidelines for prophylaxis of mucositis such as patient education, use of non-medicated saline rinses, hydration, nutritional support, infection control, non-pharmacologic and pharmacologic options that could be implemented for patients if we predict it before radiotherapy [8,12].

Most existing normal tissue complication probability (NTCP) models for RIOM depend only on clinical characteristics and dose-volume parameters [10,13]. In recent years, a number of studies have indicated relationships between the probability and severity of OM with irradiation volume and dose delivered to the oral cavity. Several Radiation Therapy Oncology Group (RTOG) clinical trials have recommended sparing oral cavity by keeping its mean dose (D_mean_) lower than 30–50 Gy (RTOG 0912, RTOG 0920, and RTOG 1216). Also, V_30Gy_ (volume receiving a dose of 30Gy or more) and V_50Gy_ of the oral cavity have been considered as independent predictors of severe OM in NPC patients treated with IMRT [14]. Moreover, other research groups found a correlation between severe OM and the volume of oral mucosa receiving 9.5–10.1 Gy/week [15,16]. In another study, patients’ age, tumor N-stage, the cycle of induction chemotherapy, and V_40_ of the oral cavity were identified as independent factors predicting severe OM [16]. It should be noted, however, the prediction of the severity of RIOM on a case-by-case basis is highly challenging [10]. Also, the strict use of the present dose-volume constraints does not prevent severe OM in some patients [17]. Taken together, no NTCP model has been developed to confidently guide clinical decision-making until now. The absence of prognostic and predictive biomarkers for radiation-related health effects is known as a major unmet clinical need in the modern RT era (*i.e.,* precision RT and personalized RT). Hence, it is essential to apply additional patient-specific and reproducible factors to develop accurate prediction models.

Recently, the advancements in the field of artificial intelligence (AI), as a broad area of computer sciences, have provided tremendous opportunities to analyze a huge amount of data, and machine learning tools, a branch of AI, have attracted extensive attention for use in diverse fields within medicine [18,19], including in radiation oncology [20,21]. Over the past decade, radiomics, as an advanced image-processing framework, has emerged to extract mineable high-dimensional data from routinely acquired radiologic images to help clinical decision support systems [22]. With the advent and development of radiomics, quantitative imaging features can be considered as biomarkers towards personalized medicine in radiation oncology [22]. Many studies have indicated that radiomics can be a valuable tool to facilitate precision diagnosis, treatment planning, and predicting outcomes [22]. In recent years, it has been shown that integrating quantitative medical imaging biomarkers into clinical and dosimetric data has improved the prediction of radiation-induced toxicities in the treatment of various cancers [19,[23], [24], [25], [26]]. Moreover, the advances in AI, principally machine learning models, have boosted the potential of the typically high-dimensional quantitative radiomics features (RFs) in predicting RT-induced toxicity [[26], [27], [28]]. For example, several studies applied machine learning approaches based on radiomic-derived features to predict both early and late radiation-induced xerostomia in HNC patients, showing promising results [29,30].

The objective of the present study was to:

• Utilize baseline computed tomography (CT)-derived radiomic features (RFs), dosimetric data, and clinical characteristics to develop prediction models for radiation-induced oral mucositis (RIOM) using a random forest classifier in head and neck cancer (HNC) patients undergoing intensity-modulated radiation therapy (IMRT).
• Investigate the added value of RFs extracted from pre-treatment CT images (specifically, CT simulation images) in comparison to dosimetric and clinical data for predicting RIOM.

To our knowledge, this is the first study to construct a prediction model for RIOM by incorporating integrated features (radiomics + dosimetric + clinical) through the application of a machine learning model.

## 2 Materials and methods

### 2.1 Patient population

Between November 2020 and August 2022, a total of 49 HNC patients treated with RT were enrolled in this prospective, single-institution study. The study was approved by the local ethics committee with the ethics approval number as IR.IUMS.REC.1400.699. Written informed consent was obtained from all the patients prior to their participation in this study.

Our inclusion criteria were as follows: (1) adult patients with an age ≥18 years; (2) a histologically proven HNC; and (3) a curative-intent treatment by (chemo)-RT. Patients with any symptoms of acute OM and salivary dysfunction before RT initiation, head and neck metastatic disease, prior history of chemotherapy or head and neck irradiation, prior history of oral surgery, dental implant, and periodontal disease were excluded. Table 1 outlines a summary of the clinical characteristics of the patients in this study.

**Table 1:** Summary of patients’ characteristics.

Patient variable	Number (%)
Sex	
**Male**	36 (73)
**Female**	13 (27)
**Age in years**	Median: 52 (Range: 22–81)
**Oral mucositis** ≥ **3**	26 (53.1)
**Oral mucositis** < **3**	23 (46.9)
**T stage**	
**T0**	3 (6.1)
**T1**	8 (16.3)
**T2**	16 (32.7)
**T3**	13 (26.5)
**T4**	9 (18.4)
**N stage**	
**N0**	27 (55.1)
**N1**	8 (16.3)
**N2**	9 (18.4)
**N3**	5 (10.2)
**Tumor site**	
**Nasopharynx**	10 (20.4)
**Oropharynx**	11 (22.5)
**Hypopharynx**	3 (6.1)
**Larynx**	5 (10.2)
**Oral cavity**	2 (4.1)
**Salivary gland**	1 (2.0)
**Nasal cavity**	8 (16.3)
**Other sites**	9 (18.4)

### 2.2 CT image acquisition

In this study, all CT simulation images were acquired in a supine position prior to RT start. For each patient, one set of CT scan image was used for modeling. CT images were acquired using a 16-slice Siemens SOMATOM Sensation scanner (Siemens Medical Systems, Erlangen, Germany). The CT acquisition parameters were 120 kVp, 200 mAs, 512 × 512 matrix size, 1.2 × 1.2 mm^2^ pixel size, and 3 mm slice thickness. However, in the image analysis, we discarded CT images with severe artifacts to prevent undesirable strong impact on the image features and analysis.

### 2.3 Radiotherapy treatment and endpoint toxicity definition

Herein, the dataset included patients with a range of head and neck primary disease sites. All patients were treated with image-guided IMRT using Varian Clinac 600C linear accelerator (Varian Medical Systems, Palo Alto, CA, USA). RT prescription dose was 60 Gy, 65 Gy or 70 Gy in 30, 33, or 35 fractions, respectively.

OM toxicity in all the patients was consistently scored according to the Common Terminology Criteria for Adverse Events (CTCAE) scale version 5.0. Herein, patients were assessed for toxicity at baseline (*i.e.,* prior to the start of RT), weekly during RT treatment course, and 8 weeks post-RT. An experienced HNC specialist trained in the use of the scoring systems was appointed to record the OM of patients. According to CTCAE v5.0, the maximum reported OM grade during follow-up, the patients were dichotomized into two groups: (1) severe OM (grade ≥3), *i.e.,* at least one grade 3 or grade 4 event at any time during follow-up; (2) and non-severe OM (grade <3). At baseline, there was no toxicity of oral mucosa in any patient.

### 2.4 Oral mucosa segmentation

**Fig. 1:**
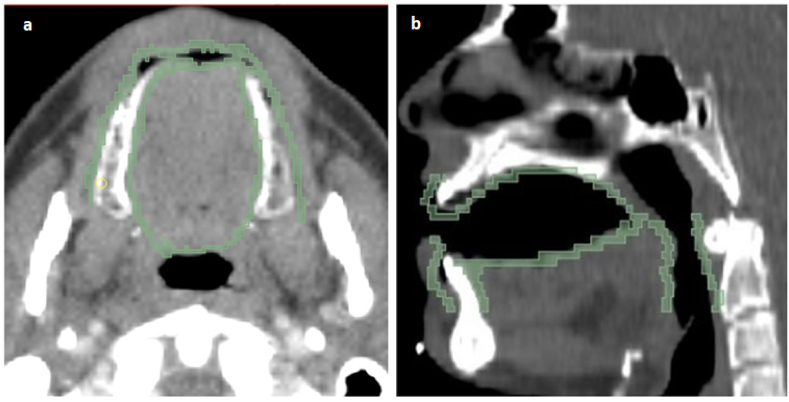
Manual segmentation of oral mucosa on CT image from a representative patient a) axial and b) sagittal view.

The planning CT images were imported into open-source 3D Slicer software version 4.10.0 [31]. Then, the oral mucosa structure was manually delineated by a junior radiation oncologist under the supervision of a senior radiation oncologist. The structures were contoured using the segmentation tool of 3D Slicer software. The mucosal surface contours (MSC) were defined as a 3 mm thick wall of tissue according to method as described by Ueno et al. [32]. The delineated MSC included mucosa surface of buccal mucosa, buccal gingiva, gingiva proper, lingual gingiva, lingual frenulum, alveolar mucosa, labial mucosa, labial gingiva, labial frenulum, mucosal surface of the floor of the mouth, mucosal surface of tongue anterior to the terminal sulcus, mucosal surface of the hard palate, and the inferior mucosal surface of the soft palate [33]. Fig. 1a and b depicts an example of delineation of the oral mucosa structure in CT images in axial and sagittal view.

### 2.5 Feature extraction

PyRadiomics toolbox [34], provided as an extension module in the 3D Slicer software, was utilized to extract the RFs from the segmented region of interest (ROI) in CT images. Herein, RFs were derived from original CT images (*i.e.,* no filter applied) as well as after wavelet transform (all combinations of applying either a high-pass or low-pass filter in each of the x-, y-, and z-axis directions). All setting parameters of PyRadiomics were default. Using the radiomics module, RFs were extracted from CT images, including shape, first-order, Gray-Level Co-occurrence Matrix (GLCM), Gray-Level Dependence Matrix (GLDM), Gray-Level Run-Length Matrix (GLRLM), Gray-Level Size-Zone Matrix (GLSZM), and Neighboring Gray-Tone Difference Matrix (NGTDM). A total of 851 RFs were extracted using the original CT images as well as after wavelet decomposition.

In addition, for every patient, 84 dosimetric parameters of oral mucosa structure were extracted, including D5–D100 (in 5 % increments), V5–V100 (in 5 % increments), D_mean_, maximum dose (D_max_), minimum dose (D_min_), and total volume for the oral mucosa. Also, for every patient, 38 clinical parameters, such as age, gender, BMI, staging, chemotherapy regimens, smoke, etc., were extracted. The detailed clinical features are shown in Supplementary Material 1, S1. In total, 851 CT RFs, 84 dose-volume features, and 38 clinical features were extracted for each patient. We have normalized our features prior to any processes by using robust scaler from sickit learn package [35].

### 2.6 Feature selection

We randomly split the dataset into the training set (n = 39) and the testing set (n = 10) with 80-20 ratio. Radiomics, dosimetric, and clinical features extracted from the training dataset were utilized for the feature selection procedure. Herein, the maximum Relevance Minimum Redundancy (mRMR) algorithm was used as a feature selection method [35]. The mRMR, an entropy-based feature selection method, was applied to rank all extracted feature groups separately based on their relevance to RIOM, eliminating the redundant and irrelevant features. All RFs were normalized using Z-score.

### 2.7 Classifier model

**Fig. 2:**
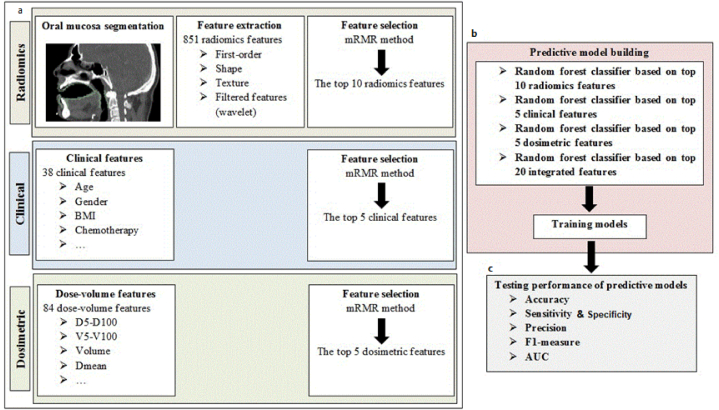
Workflow of the proposed models for prediction of oral mucositis a) features extraction b) number of selected features for each model c) testing performance for predictive models.

In this study, a two-class (binary) classification approach using machine learning methods was employed to compare patients with and without OM. Following feature selection, predictive machine learning models were created using radiomic, dosimetric, and clinical features either individually or in combination. The workflow of the proposed predictive models is illustrated in Fig. 2. Fig. 2a shows the features extraction in each model and Fig. 2b and c shows the brief about the number of selected features and the testing performance for predictive models respectively. The random forest algorithm was utilized to train the models using the training dataset. The selected features were used as covariates (X), while the presence or absence of OM was used as the dependent variable (Y) in the random forest algorithm. The random forest algorithm was implemented using the Python scikit-learn machine learning package (version 0.20.4) to build the prediction models [35].

### 2.8 Performance evaluation

The performance of the proposed prediction models was assessed using the testing dataset (n = 10). The area under the receiver operating characteristic (ROC) curve (AUC) was used to quantify the predictive values of the models. In addition, the performance of predictive models was also evaluated using accuracy, sensitivity, specificity, precision, and F1-measure calculated based on the confusion matrices as follows:

$$\text{Accuracy}=\frac{\text{TP}+\text{TN}}{\text{TP}+\text{FP}+\text{TN}+\text{FN}}$$

$$\text{Sensitivity}=\frac{\text{TP}}{\text{TP}+\text{FN}}$$

$$\text{Specificity}=\frac{\text{TN}}{\text{TN}+\text{FP}}$$

$$\text{Precision}=\frac{\text{TP}}{\text{TP}+\text{FP}}$$

$$F1-\text{measure}=2\times \frac{\text{Precision}\times \text{Sensitivity}}{\text{Precision}+\text{Sensitivity}}$$

where: TP: True Positive. FP: False Positive. TN: True Negative, and FN: False Negative.

## 3 Results

### 3.1 Study subjects

Table 1 outlines a summary of the clinical characteristics of the patients. A total of 49 HNC patients with a median age of 52 years (range, 22–81 years) were included in this study, of whom 36 (73 %) patients were male and 13 (27 %) were female. As shown in Table 1, RIOM (grade ≥3) was experienced in 26 of 49 patients. Most patients had nasopharynx cancer, followed by oropharynx and nasal cavity cancers (Table 1).

### 3.2 Features selected with mRMR feature selection method

With the participation of radiation oncologists, the top mRMR-ranked features were selected to train random forest classifier, because using fewer features can efficiently prevent overfitting. After application of the mRMR method, the top 10 RFs, the top 5 dose-volume features, and the top 5 clinical features were selected. The top selected feature (radiomics, clinical, dosimetric) after feature selection are given in Table 2. The class of the radiomic features are also shown in this table.

**Table 2:** The top selected feature (radiomics, clinical, dosimetric) after feature selection.

Rank	Top ranked radiomics features (Feature Class)	Top ranked Clinical features	Top ranked Dosimetric features
**1**	Mean.2 (First Order)	Number of chemotherapy course cisplatine (w.RT)	D60 % (Gy)
**2**	Imc1 (GLCM)	Flossing teeth (w.RT)	D65 cc (Gy)
**3**	Skewness.2 (First Order)	Number of chemotherapy course erbitax (w.RT)	D100 % (Gy)
**4**	LongRunHighGrayLevelEmphasis.4 (GLRLM)	Number of chemotherapy course cetuxib (w.RT)	V75 (%)
**5**	Skewness.7 (First Order)	Using of magic gelclair	D50 cc (Gy)
**6**	Idmn.4 (GLCM)	–	–
**7**	Mean.6 (First Order)	–	–
**8**	LowGrayLevelZoneEmphasis.8 (GLSZM)	–	–
**9**	Correlation.3 (GLCM)	–	–
**10**	MCC.4 (GLCM)	–	–

**Fig. 3:**
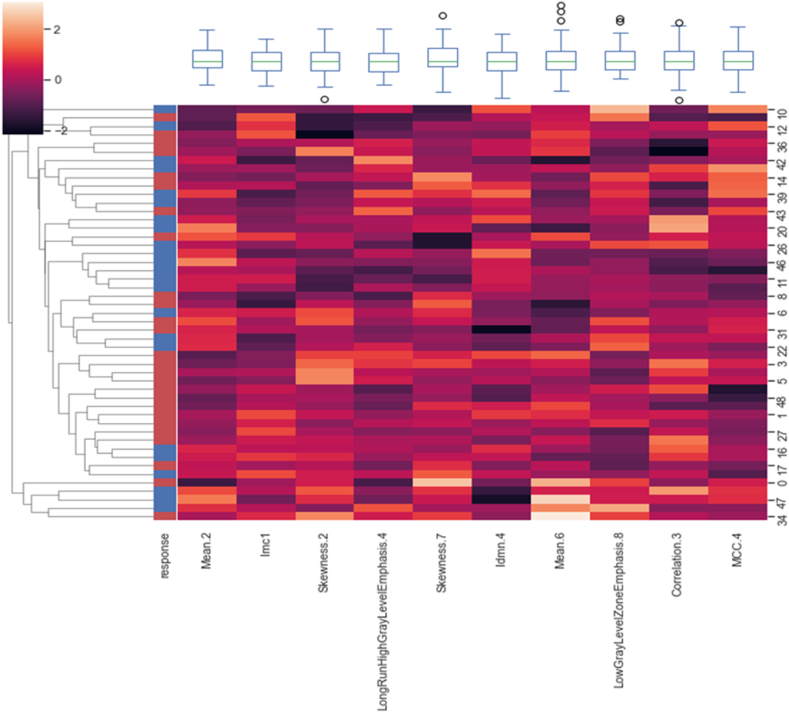
Heatmap showing the clustering of 10 selected radiomic features.

**Fig. 4:**
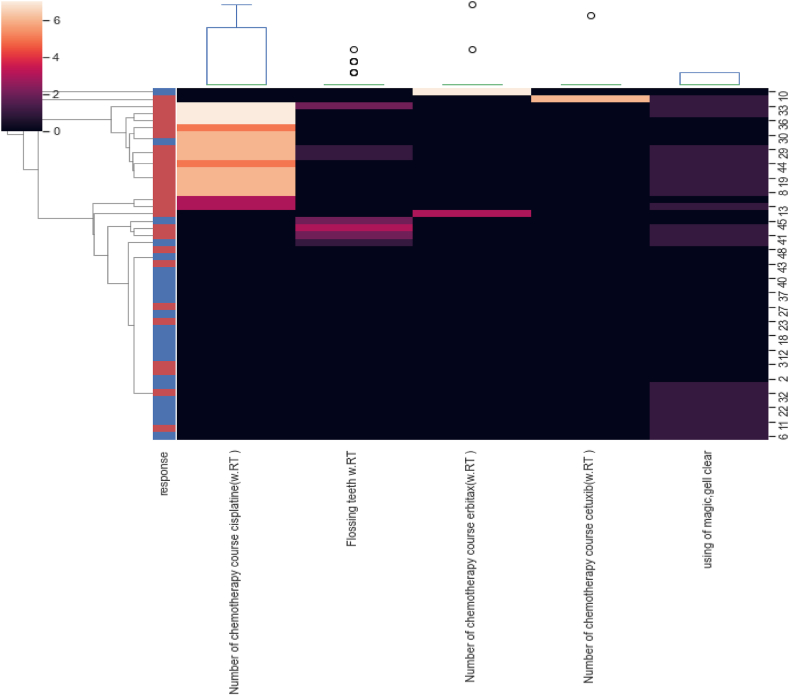
Heatmap showing the clustering of 5 selected clinical features.

**Fig. 5:**
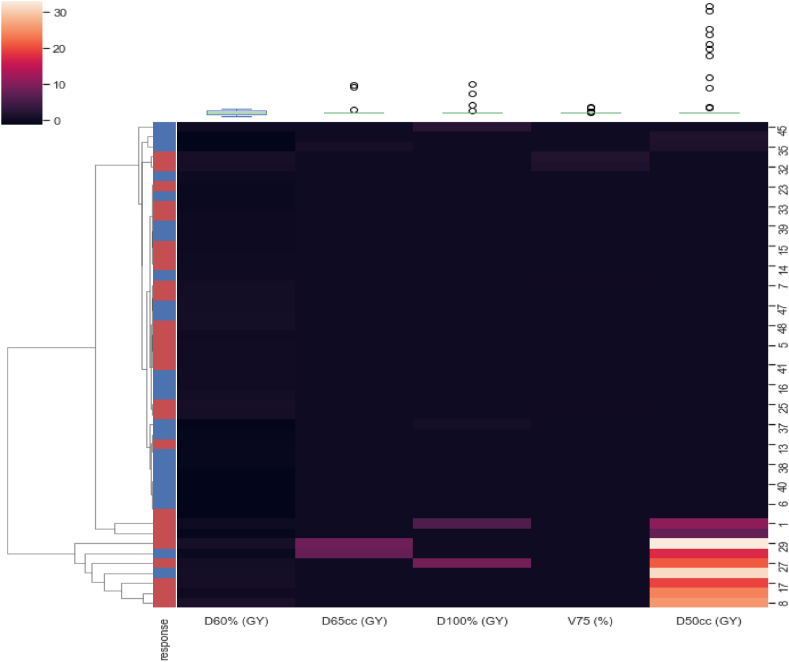
Heatmap showing the clustering of 5 selected Dosimetric features.

Fig. 3, Fig. 4, Fig. 5 shows the heatmap illustrating the clustering of selected radiomic, clinic, and dosimetric robust features respectively which have the highest correlation with mucositis.

**Fig. 6:**
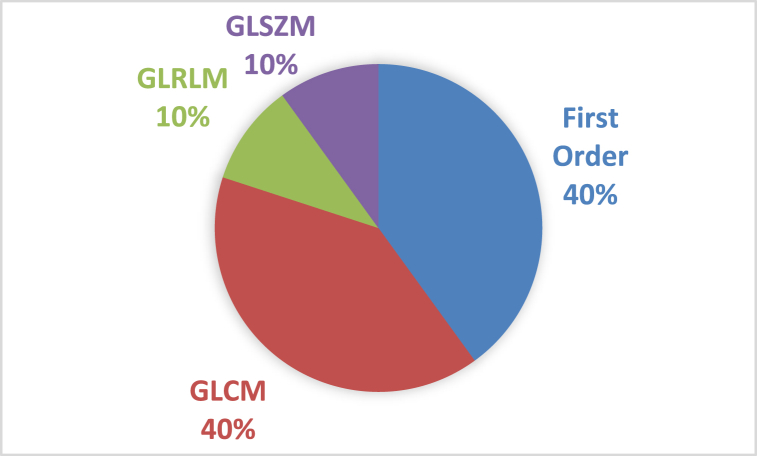
Percentage distribution of selected Radiomic feature classes.

Fig. 6 shows the percentage of each class of selected radiomic features. As can be seen, the first-order and GLCM classes are the most abundant among all selected feature classes.

### 3.3 Oral mucositis prediction models

**Fig. 7:**
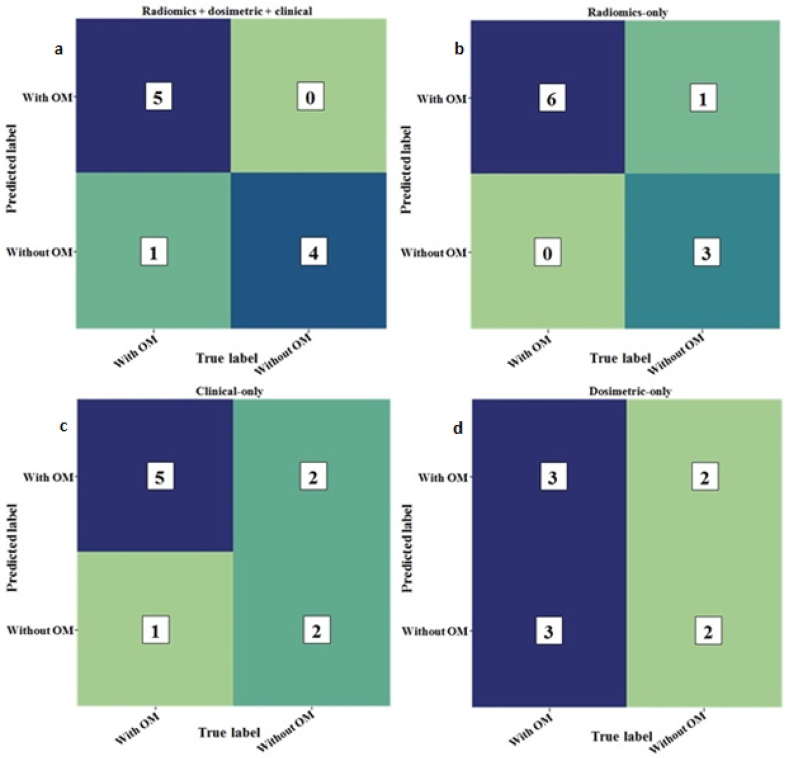
Confusion matrices for the four prediction models of oral mucositis a) Radiomics + dosimetric + clinical, b) only radiomics, c) only clinical and d) only dosimetric model.

**Fig. 8:**
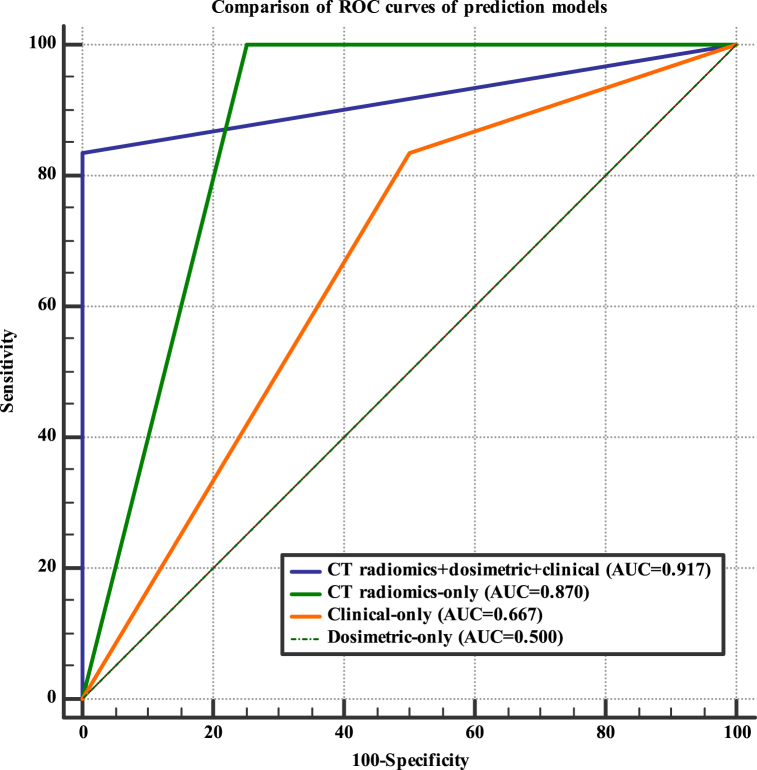
Receiver operating curve (ROC) analysis for predicting oral mucositis using the testing dataset.

A random forest classifier algorithm was trained both on the three groups of features selected individually (CT RFs, dose-volume features, and clinical features) and jointly. The performances of the various prediction models for the development of OM were assessed on the unseen testing dataset (n = 10; 6 with OM and 4 without OM), as shown in Table 3. Fig. 7a, b, c and d depicts the classification performances of the acute OM predictive models in the form of confusion matrices for Radiomics + dosimetric + clinical, only radiomics, only clinical and only dosimetric model respectively. Fig. 8 illustrates the ROC curve of prediction models for OM. The prediction model of OM based on random forest classifier using the top 20 integrated features (including top 10 CT RFs, 5 dose-volume features, and 5 clinical features) achieved the highest performance with an AUC of 0.917, an accuracy of 90.0 %, a sensitivity of 83.0 %, a precision of 100.0 %, and a F1-measure of 91.0 %, as observable in Table 3. The CT RFs-based model achieved the best performance compared to dose-volume features or clinical features alone, with an AUC of 0.87. The model developed using dosimetric features alone provided the poorest performance for predicting OM, as outlined in Table 3.

**Table 3:** The performance of predictive models for development of radiation-induced oral mucositis on the testing dataset (n = 10).

Prediction models	AUC	Sensitivity	Specificity	Accuracy	Precision	F1-measure
**Radiomics + dosimetric + clinical**	91.7 %	83 %	100 %	90 %	100 %	91.0 %
**Radiomics-only**	87.0 %	100 %	75 %	90 %	86 %	92 %
**Clinical-only**	66.7 %	83 %	50 %	70 %	71 %	77 %
**Dosimetric-only**	50.0 %	50.0 %	50.0 %	50 %	60 %	55 %

## 4 Discussion

RT is known as the backbone of the multimodal therapy in HNC [2]. However, RT for HNC is often associated with significant side effects, including OM [7]. OM is the most common toxicity of RT for HNC patients and is owing to damage to the irradiated basal epithelial cells of the oral mucosa with a relevant influence on the patient s’ QOL [7,36]. Hence, accurately predicting OM can support clinical decision-making for early intervention and planning personalized treatment. Numerous studies have shown that radiomics has great potential to provide useful quantitative imaging features for clinical prediction [[37], [38], [39]]. In recent years, radiomics has been applied in different fields of HNC RT, including survival prediction, treatment response assessment, tumor segmentation, diagnosis and identification, prognostication, staging, treatment planning, and toxicity prediction [24,40]. In addition, recent advances in AI have provided great tools with capabilities to analyze a massive amount of data, which in turn, further increasing the potential of radiomics. Nevertheless, it has previously been reported that it would be best to employ RFs along with dosimetric and clinical features, rather than in isolation, to boost the power of predictive models [41]. To this end, the purpose of this study was to investigate whether adding baseline CT-derived RFs to dose-volume and clinical data will improve the performance of machine learning model in predicting OM in HNC patients treated with curative IMRT. Hence, the performance of the prediction models under four different scenarios was evaluated: 1) only CT RFs, 2) only clinical features, 3) only dose-volume features, and 4) combination of CT radiomics, clinical, and dose-volume features. To our knowledge, this is the first study to incorporate pre-treatment CT RFs with dosimetric and clinical features to establish a prediction model for RIOM.

Based on our data, it is evident that the utilization of integrated features (CT radiomics + dosimetric + clinical) yielded the highest performance in predicting radiation-induced oral mucositis (OM). While the random forest classifier, when applied solely to CT radiomic features, demonstrated good predictive capability in our study, the inclusion of dosimetric and clinical features alongside the radiomic features further enhanced the performance of the prediction model. In essence, the radiomic signature derived from pre-treatment CT images (specifically, CT simulation) exhibited strong predictive power concerning both dose-volume and clinical features. As indicated in Table 3 and Fig. 7, Fig. 8, the machine learning model based on radiomic features alone achieved AUC, accuracy, sensitivity, precision, and F1-measure values of 87.0 %, 90.0 %, 100.0 %, 86.0 %, and 92.0 %, respectively. The augmentation of dosimetric and clinical information contributed to an improvement in AUC (91.7 %) and precision (100.0 %) values. Our findings align with previous studies [23,42], demonstrating that the incorporation of baseline imaging RFs with dose-volume and clinical data improves the accuracy of machine learning models in predicting radiation-induced xerostomia compared to clinical or dose-volume features alone. In contrast to the findings of Sheikh et al.'s study, our present study identified different top clinical features for mucositis prediction, as shown in Table 2. The significant clinical features in our study encompassed chemotherapy regimens and personal oral and dental care habits, while other clinical parameters did not exhibit notable importance in relation to mucositis. This finding holds potential implications for healthcare professionals in terms of treatment management and mucositis prevention strategies. Certainly, incorporating additional clinical parameters into the model, such as blood factors, genetics, eating habits and etc., could potentially yield different results in mucositis prediction. By expanding the range of clinical variables considered, it is possible to uncover new insights and enhance the accuracy of the prediction model for mucositis. Therefore, exploring and including such parameters in future studies may provide a more comprehensive understanding of the factors contributing to mucositis prediction.

In a study conducted by Liu et al., they aimed to develop a nomogram for predicting severe oral mucositis in patients with nasopharyngeal carcinoma undergoing IMRT radiotherapy. Similar to our study, they also identified the cycle of induction chemotherapy as a potential predictor of severe oral mucositis [43]. However, there were some differences between their findings and our study. Liu et al. found that age and N stage of the primary tumor were also predictive of oral mucositis, which contrasts with our findings. This difference might be attributed to the fact that they did not consider other factors such as oral and dental care habits of the patients in their analysis.

The inclusion or exclusion of certain factors in the predictive models can lead to variations in the identified predictors of oral mucositis. It highlights the importance of considering multiple factors and conducting comprehensive analyses to gain a more complete understanding of the predictors of oral mucositis in specific patient populations. From our data, it is obvious that the models developed using dosimetric or clinical features alone performed poorly for the prediction of OM. Nevertheless, the results obtained from modeling based solely on radiomic features of the CT image are comparable to the results obtained from modeling using the combined model. This suggests that the radiomic features alone carry significant predictive power for mucositis. While the inclusion of additional clinical and dosimetric parameters may provide valuable insights in our prediction. In the light of our results, it seems in fact that the texture of the organ at risk (*i.e.,* oral mucosa structure) before radiotherapy, is a strong predisposing risk factor for the development of OM. In other words, the texture of the oral mucosa structure can be a personal risk factor for developing OM. In this study, the mRMR feature selection method was employed, and as a result, the top 10 ranked CT radiomic features (RFs) primarily consisted of radiomic texture features. These texture features encompassed various categories, including First Order, GLCM, GLSZM, and GLRLM, which quantitatively capture textural characteristics. Notably, the selected list of features in Table 2 did not include any shape-based features.

From a patient’s perspective, RIOM is one of the most severe patient-reported toxicity during HNC RT. From a clinical point of view, severe OM is of special importance because it impairs RT course and patients’ QOL [44]. Accurate and early identification of patients at high-risk of developing severe OM is critical because can provide early intervention and disease prevention, thereby possibly mitigating the incidence and severity of OM in HNC patients during RT. Over the last decade, numerous studies have employed radiomics for a myriad of applications in radiation oncology, including treatment toxicity prediction [22,45]. Also, fewer studies have conducted to integrate radiomics with AI to increase its potential [46,47]. In radiation therapy, using the integrated RFs and machine learning models with dosimetric and patient-specific features can facilitate clinical decision-making and personalized RT. Herein, we applied CT RFs, dosimetric and clinical data to construct prediction models for OM. Although machine learning model based on the integrated features (radiomics + dosimetric + clinical) achieved encouraging results, further study will be required to prospectively validate our results prior to clinical translation. Currently, our proposed prediction model can possibly be considered as a computer-assisted clinical decision support tool to aid physicians in predicting RIOM in HNC patients.

It is worthwhile to mention that single-time point pre-treatment CT images were used to extract RFs in this study, demonstrating that baseline CT-derived RFs-based machine learning model show promising performance in predicting OM in HNC RT. Herein, we did not consider the temporal kinetics of RFs during RT. More recently, studies have shown that longitudinal analysis of radiation-induced changes of CT RFs can improve the identification of individuals at high risk ahead of developing RT-induced toxicity [[48], [49], [50]]. Therefore, further research is warranted to investigate whether the temporal kinetics of RFs during RT can improve the performance of our proposed machine learning classifier trained on the integrated features.

The proposed machine learning model based on the combination of features demonstrated promising performance in predicting OM. However, it is important to acknowledge the inherent limitations of this study. Firstly, the study was conducted at a single center with a relatively small number of patients, which may restrict the generalizability of the proposed prediction models. Therefore, future research should involve validation on larger-scale, multi-institutional studies to enhance the robustness of the models. Additionally, while the performance of the models was assessed on an internal unseen testing dataset, further investigation is necessary to validate the proposed models using an independent external dataset. This will help ensure the reliability and applicability of the models in real-world clinical settings.

## 5 Conclusion

In conclusion, this study highlights the potential of combining baseline CT-based radiomic features with dosimetric and clinical features for predicting OM in HNC radiotherapy. The integration of these features offers a more accurate clinical decision-making support tool for clinicians treating HNC patients. The random forest classifier, utilizing the top 20 integrated features (10 CT radiomics + 5 dosimetric + 5 clinical features), demonstrated a favorable performance with an AUC value of 0.917. Moreover, while the prediction model based solely on CT radiomic features showed predictive capability, incorporating integrated features resulted in improved predictions with enhanced clinical utility. To validate the predictive ability of the proposed models and facilitate their integration into routine clinical practice, further studies involving multicenter, large-scale, prospective cohorts will be essential.

## Funding

This study has received funding by 10.13039/100012021Iran University of Medical Sciences, Tehran, Iran, and grant number is 20801.

## Research involving human participants and/or animals

This study involved human participants, and it was conducted considering ethic responsibilities according to the World Medical Association and the Declaration of Helsinki.

## Ethical approval

The study was approved by the ethics committee of Iran University of Medical Sciences, Tehran, Iran. Ethics No. is IR.IUMS.REC.1400.699.

## Informed consent

Informed consent was obtained from all individual participants prior to their inclusion in the study.

## CRediT authorship contribution statement

**Razieh Agheli:** Data curation. **Zahra Siavashpour:** Data curation. **Reza Reiazi:** Software. **Samira Azghandi:** Data curation. **Susan Cheraghi:** Writing – review & editing, Writing – original draft, Supervision. **Reza Paydar:** Writing – original draft, Supervision, Conceptualization.

## Declaration of competing interest

The authors declare that they have no known competing financial interests or personal relationships that could have appeared to influence the work reported in this paper.
