# Supplementary material for: Predicting severe radiation-induced oral mucositis in head and neck cancer patients using integrated baseline CT radiomic, dosimetry, and clinical features: A machine learning approach
Source: Heliyon. 2024 Jan 24;10(3):e24866. doi: 10.1016/j.heliyon.2024.e24866 (PMC10839875; doi:10.1016/j.heliyon.2024.e24866)
Supplement: Multimedia component 1 [file mmc1.docx]

**S1:** The extracted clinical features

**Table S1:** The extracted clinical features

|  | **Clinical feature** |
| --- | --- |
| 1 | Age |
| 2 | Sex |
| 3 | BMI before radiotherapy |
| 4 | BMI after radiotherapy |
| 5 | Smoking before radiotherapy |
| 6 | Smoking with radiotherapy |
| 7 | Brushing teeth with radiotherapy |
| 8 | Flossing teeth with radiotherapy |
| 9 | Using of banzidamyn and bicarbonate |
| 10 | Using of magic gelclair |
| 11 | T stage |
| 12 | N stage |
| 13 | Performance status |
| 14 | Chemotherapy |
| 15 | Number of chemotherapy course Taxol + carboplatine before radiotherapy |
| 16 | Number of chemotherapy course Taxol + carboplatine with radiotherapy |
| 17 | Number of chemotherapy course cisplatine before radiotherapy |
| 18 | Number of chemotherapy course cisplatine with radiotherapy |
| 19 | Number of chemotherapy course cisplatine + 5 fu before radiotherapy |
| 20 | Number of chemotherapy course cisplatine + 5 fu with radiotherapy |
| 21 | Number of chemotherapy course cisplatine + etopzid before radiotherapy |
| 22 | Number of chemotherapy course cisplatine + etopzid with radiotherapy |
| 23 | Number of chemotherapy course carboplatine + gemzar before radiotherapy |
| 24 | Number of chemotherapy course carboplatine + gemzar with radiotherapy |
| 25 | Number of chemotherapy course carboplatine before radiotherapy |
| 26 | Number of chemotherapy course carboplatinew before radiotherapy |
| 27 | Number of chemotherapy course cisplatine + gemzar before radiotherapy |
| 28 | Number of chemotherapy course cisplatine + gemzar with radiotherapy |
| 29 | Number of chemotherapy course cetuxib before radiotherapy |
| 30 | Number of chemotherapy course cetuxib with radiotherapy |
| 31 | Number of chemotherapy course erbitax before radiotherapy |
| 32 | Number of chemotherapy course erbitax with radiotherapy |
| 33 | Number of chemotherapy course carboplatine + erbitax before radiotherapy |
| 34 | Number of chemotherapy course carboplatine + erbitax with radiotherapy |
| 35 | Number of chemotherapy course cisplatine + Taxol + erbitax before radiotherapy |
| 36 | Number of chemotherapy course cisplatine + Taxol + erbitax with radiotherapy |
| 37 | Number of chemotherapy course gemzar before radiotherapy |
| 38 | Number of chemotherapy course gemzar with radiotherapy |
